# *In Vivo* Non-Thermal, Selective Cancer Treatment With High-Frequency Medium-Intensity Focused Ultrasound

**DOI:** 10.1109/access.2021.3108548

**Published:** 2021-08-27

**Authors:** YONGKUI TANG, LENG-YING CHEN, AILIN ZHANG, CHUN-PENG LIAO, MITCHELL ERIC GROSS, EUN SOK KIM

**Affiliations:** 1 Department of Electrical and Computer Engineering, University of Southern California, Los Angeles, CA 90089, USA; 2 Lawrence J. Ellison Institute for Transformative Medicine, University of Southern California, Los Angeles, CA 90064, USA

**Keywords:** High-frequency focused ultrasound, *in vivo* experiment, non-invasive therapy, selective cancer treatment, self-focusing acoustic transducers (SFAT), ultrasound therapy

## Abstract

Focused ultrasound (FUS) has proven its efficacy in non-invasive, radiation-free cancer treatment. However, the commonly used low-frequency high-intensity focused ultrasound (HIFU) destroys both cancerous and healthy tissues non-specifically through extreme heat and inertial cavitation with low spatial resolution. To address this issue, we evaluate the therapeutic effects of pulsed (60 Hz pulse repetition frequency, 1.45 ms pulse width) high-frequency (20.7 MHz) medium-intensity (spatial-peak pulse-average intensity I_SPPA_ < 279.1 W/cm^2^, spatial-peak temporal-average intensity I_SPTA_ < 24.3 W/cm^2^) focused ultrasound (pHFMIFU) for selective cancer treatment without thermal damage and with low risk of inertial cavitation (mechanical index < 0.66), in an *in vivo* subcutaneous B16F10 melanoma tumor growth model in mice. The pHFMIFU with 104 *μ*m focal diameter is generated by a microfabricated self-focusing acoustic transducer (SFAT) with a Fresnel acoustic lens. A three-axis positioning system has been developed for automatic scanning of the transducer to cover a larger treatment volume, while a water-cooling system is custom-built for dissipating non-acoustic heat from the transducer surface. Initial testing revealed that pHFMIFU treatment can be applied to a living animal while maintaining skin temperature under 35.6 °C without damaging normal skin and tissue. After eleven days of treatment with pHFMIFU, the treated tumors were significantly smaller with large areas of necrosis and apoptosis in the treatment field compared to untreated controls. Potential mechanisms of this selective, non-thermal killing effect, as well as possible causes of and solutions to the variation in treatment results, have been analyzed and proposed. The pHFMIFU could potentially be used as a new therapeutic modality for safer cancer treatment especially in critical body regions, due to its cancer-specific effects and high spatial resolution.

## I. INTRODUCTION

Focused ultrasound (FUS) is a powerful and effective tool for non-invasive therapy. With the energy of ultrasound focused onto a small volume of tissue that can be deep inside the body, the treatment efficacy and precision is greatly enhanced with less side effects compared to less focused radiation [1]. For example, high-intensity focused ultrasound (HIFU) has demonstrated good therapeutic effects in the treatment of tumors [2] in the prostate [1], [3], pancreas [4], breast [5], and brain [6]. In most of these applications, low-frequency (<4 MHz) focused ultrasound of high intensity (with spatial-peak pulse-average intensity I_SPPA_ usually ranging from 1,000 to 10,000 W/cm^2^) induces rapid heating in tissue [7], raising its temperature above 60 °C, to cause irreversible cell damage (coagulative necrosis) [8]. Apart from direct heating, HIFU, especially with high pressure (>10 MPa), short pulse width (<20 *μ*s) at a low duty cycle (<1%) [9], [10], has been shown to induce inertial acoustic cavitation, in which submicron/micron-sized gas bubbles form from cavitation nuclei and collapse rapidly after growth, causing destructive mechanical damage from shock waves or high-speed microjets [11]. Although both heat and cavitation effectively destroy tumor cells, nearby normal tissue in the target area is also affected by the non-specific damage during the treatment, causing unwanted side effects, or even resulting in loss of bodily function on some critical parts of the body [12]–[15]. As a result, most clinical treatment with HIFU must operate under the guidance of external imaging methods [4] such as magnetic resonance imaging (MRI) [1], [5], [6] or ultrasound imaging [9], greatly increasing the cost and complexity of the procedure. Moreover, at low frequencies, the focal volume of focused ultrasound is large due to long acoustic wavelength [16], and the heating and inertial cavitation effects combined with beam inhomogeneity may make the actual lesion region extend beyond the desired region or even the intended focal region [17]–[19], resulting in a poor spatial resolution of the treatment. For example, a 1-MHz HIFU transducer has a focal diameter and depth of focus of 2 and 8 mm, respectively, but the actual lesion size could be 10 mm × 16 mm [17]. As a result, even with imaging guidance, delivery of a potentially therapeutic dose of focused ultrasound to invasive cancer cells near critical structures such as neural circuits and blood vessels in the brain [6], [20] is still highly challenging. Thus, a high-resolution ultrasound treatment that could selectively destroy cancer cells without damaging benign cells would be highly desirable.

For selective treatment of cancer, strategies which may include agents such as gold or magnetic nanoparticles [21] and intravenously injected microbubbles [22] have been used to increase cancer cells’ sensitivity to ultrasound treatment in experiments involving monolayer co-cultures (of normal and cancer cells) and an *in vivo* murine model, respectively. However, as the mechanisms and potential risks remain unclear, the addition of these agents may bring other undesirable complications to the treatment.

Without additional agents, selective cancer treatment with ultrasound alone has been demonstrated *in vitro* with low-intensity ultrasound without relying on temperature rise. For example, experiments with monolayer cell cultures showed that, compared to benign cells, some types of malignant cells were much more sensitive to the damaging effect from two to four minutes of exposure to low intensity (0.33 W/cm^2^) continuous-wave (CW) ultrasound at 2 MHz [23] and 20 kHz [24]. In another cell suspension model, pulsed ultrasound of low frequency (0.50–0.67 MHz) and low intensity (spatial-peak temporal-average intensity I_SPTA_ < 5 W/cm^2^, peak negative pressure < 1.2 MPa) demonstrated specific killing effect on cancer cells suspended in phosphate-buffered saline (PBS) when the pulse duration was longer than 20 ms, while most healthy cells remained undamaged [25]. However, the treatment efficacy dropped significantly when the cells were suspended in more rigid tissue-mimicking media such as agarose and acrylamide gels.

Compared to low-frequency ultrasound, high-frequency focused ultrasound has better targeting precision due to its smaller focal volume. For example, 20-MHz HIFU transducers with small depth of focus (1.1–1.7 mm) have been used in a recent clinical study to treat early-stage actinic keratosis (AK) and skin cancers, where the diameter (2–3 mm) and thickness (1–2 mm) of the tumors are small, achieving a cure rate of 97% [26]. However, since the treatment relies on HIFU-induced heat, it causes pain and inflammation, and is essentially non-selective. Previously, using pulsed high-frequency (18 MHz) low-intensity (I_SPPA_ < 15.14 W/cm^2^) focused ultrasound generated by microfabricated self-focusing acoustic transducers (SFAT), we demonstrated selective cytolysis on both monolayers [27] and three-dimensional (3D) spheroids [28] of cancer cells with high spatial resolution of 100 and 160 *μ*m, respectively. In both cases, we found that the acoustic intensity thresholds (AIT) for cytolysis of cancerous cells are substantially lower than those for benign cells, likely due to less organized actin cytoskeletal pattern (known to be associated with decreased cell stiffness) compared to benign cells [27]. Utilizing such a difference through keeping treatment acoustic intensity higher than the AIT of cancer cells and lower than that of normal cells, we successfully destroyed cancer cells without harming benign cells.

The actual tissue environment differs greatly from the artificial cell cultures, potentially resulting in different treatment efficacy of FUS between *in vivo* and *in vitro* experiments. Thus, to further confirm the effectiveness of this non-thermal selective cancer treatment with high-frequency focused ultrasound, we have developed an SFAT along with a treatment system for *in vivo* treatment of B16F10 subcutaneous melanoma tumors in mice.

## II. THE TRANSDUCER AND THE TUMOR TREATMENT SYSTEM

### A. TRANSDUCER DESIGN AND FABRICATION

**FIGURE 1.**
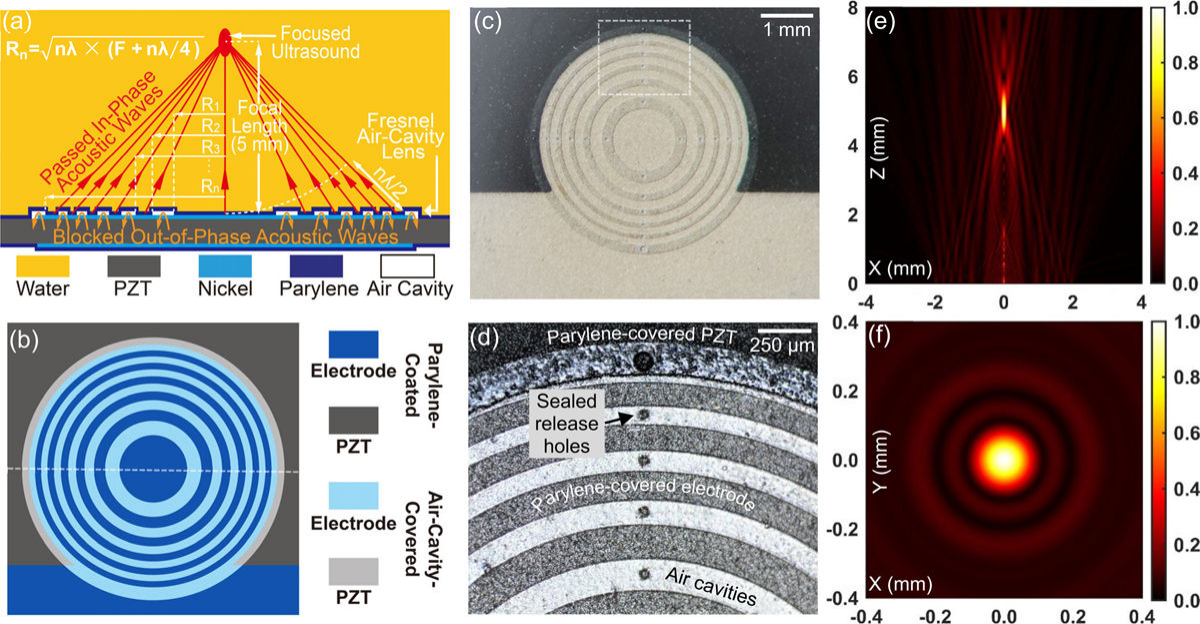
(a) Cross-sectional (across the dashed line in (b)) diagram of the transducer, showing how the Fresnel air-cavity acoustic lens focuses ultrasound by blocking destructively interfering acoustic waves. (b) Top-view diagram of the transducer showing the relative positions of the top electrode, air-cavity rings, and Parylene-coated regions. (c) Top-view photo of a fabricated SFAT before wires are soldered. (d) Microscope photo of part of the transducer (the dashed rectangular area in (c)), showing parts of five air-cavity rings with sealed release holes on the top electrode. The outermost air-cavity ring (top one in photo) covers part of the electrode and PZT. (e) (f) FEM-simulated normalized acoustic pressure (e) on the central vertical plane and (f) on the lateral focal plane (at Z = 5 mm), with same color bar scale but different dimension scales.

**FIGURE 2.**
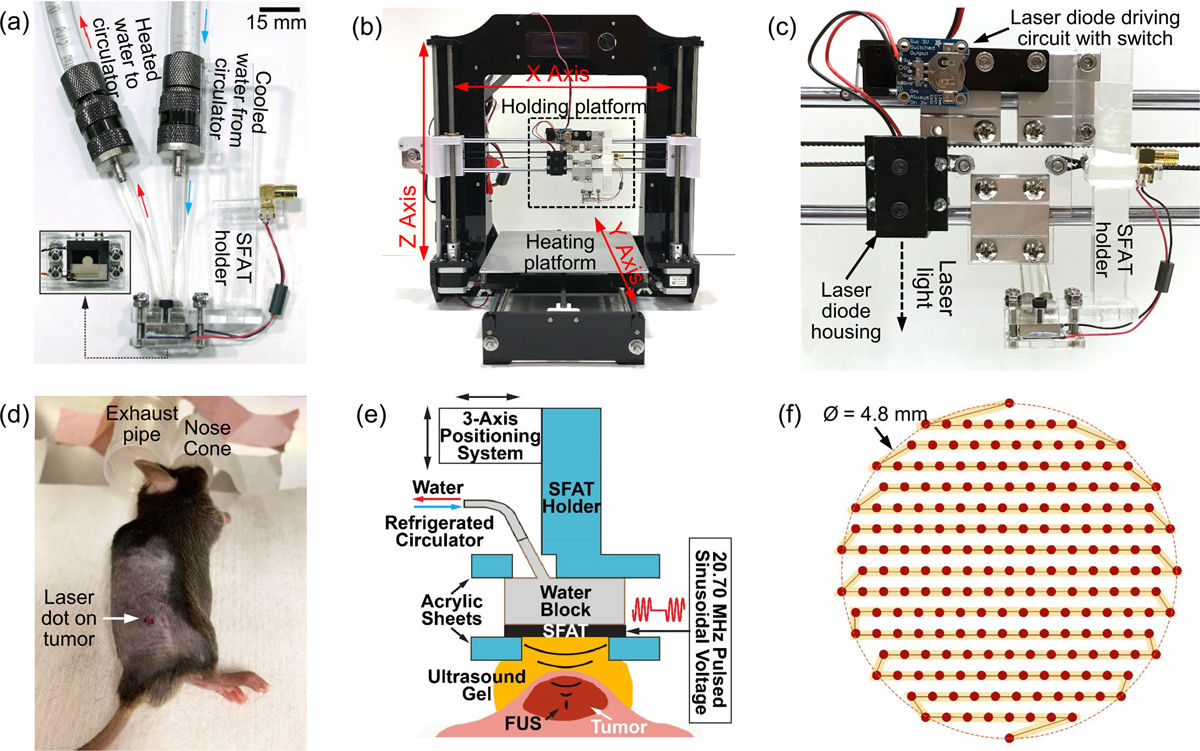
Photos of (a) a packaged SFAT on an acrylic holder, with soldered electrical wires connected to an SMA adapter for electrical connection and a hollow water-cooling block in a close-loop water-cooling system to dissipate heat from the transducer surface (Inset: front-view of the transducer clamped on the holder); (b) the 3D-printer-modified three-axis positioning system with a holding platform with the transducer attached and a heating platform where the mouse will be placed, showing how the movement in each axis is realized; (c) a close-up view of the dashed rectangular area in (b) showing the transducer holder on the right, and the laser diode in the black housing with its driving circuit on the left, all held by the movable holding platform with screws; (d) a mouse lying on the heating platform of the positioning system, anesthetized by isoflurane gas coming from an anesthesia nose cone where its nose is placed (as excessive gas is drawn away to the large exhaust pipe nearby), with a red laser dot aligned to the center of its tumor (highlighted with a dark skin marker). (e) Side-view cross-sectional diagram of the *in vivo* tumor treatment setup on mice. (f) Top-view diagram of the circular raster scan pattern of the transducer covering a circle of 4.8 mm diameter (dotted circle), showing the scan route (yellow-highlighted solid line) with 204 treatment spots (red dots). The width of the yellow line and the diameter of small solid circles are drawn in scale to indicate the focal size of the transducer, showing the actual treated area.

The SFAT (Figure 1a to 1d) used for tumor treatment consists of two parts, an ultrasonic sound source to generate ultrasound waves and an acoustic lens for focusing them. The sound source is a 1-mm-thick PZT-4 sheet (DL-47, DeL Piezo Specialties LLC) sandwiched by its top and bottom circular silver electrodes overlapping with each other in the chip center. For electrical connections, the top and bottom circular electrodes are extended into two non-overlapping rectangular soldering pads on different halves of the chip (Figure 1b and 1c), where electrical wires are soldered (Figure 2a). When sinusoidal voltage signals of 20.7 MHz are applied onto the electrodes through the soldered wires, the PZT sheet vibrates at its 9^th^ harmonic thickness-mode resonant frequency to generate ultrasound waves of 20.7 MHz, which are focused through a microfabricated Fresnel acoustic lens on the top electrode. The lens is made of Parylene-sealed annular-ring air cavities alternating with non-air-cavity regions with Parylene uniformly coated on the electrode (Figure 1a and 1b). The radii of the ring boundaries are designed to form Fresnel half-wavelength bands (FHWB) [29] for a focal length of 5 mm in water, so that the path-length difference between two adjacent ring boundaries to the designed focal point (5 mm above the center of the PZT’s top surface) is half-wavelength (Figure 1a). With this design, the acoustic waves coming from non-air-cavity regions (including the center circle and the outside rings) can propagate through the Parylene layer, and arrive at the focal point partially in-phase (with phase difference < *π*) to interfere constructively and generate focused ultrasound. The waves generated in air-cavity-ring regions (that would have contributed to destructive interference at the focal point), on the other hand, are almost completely blocked by the air cavities due to the large mismatch between the acoustic impedances of air (0.4 kRayl) and solid (over 1 MRayl) [38].

The design parameters of the transducers are summarized in Table 1. The high operating frequency is chosen for keeping the frequency similar to those used in our previous successful *in vitro* selective tumor treatment [27], [28]. At higher frequencies, the acoustic waves undergo more attenuation as they travel through the tissues compared to the low-frequency cases [30]. However, since much lower acoustic intensity is needed for the treatment, attenuation is not a concern as it can be compensated through increasing the acoustic power. A thick PZT substrate operating at the 9^th^ harmonic frequency rather than a nine-times-thinner PZT operating at its fundamental frequency is chosen due to the mechanical sturdiness of the thicker PZT for easy handling and packaging. Although at the 9^th^ harmonic frequency, the quality factor [31] and electromechanical coupling coefficient of the PZT are lower with higher mechanical and dielectric losses [32] than those at the fundamental frequency, the generated acoustic pressure is enough for this application, as shown in the measurement results mentioned in section III-A. The relatively short focal length is chosen since the target tumor in this treatment is right beneath the skin, and a long focal length is not needed. However, for applications where a deeper focus is necessary, the focal length can be increased to tens of centimeters at the cost of a larger device size, simply through redesigning the Fresnel ring patterns. To reduce unwanted dielectric heating (which is proportional to the PZT’s loss tangent [33]) during the transducer operation, PZT-4 instead of commonly used PZT-5A is selected due to its lower loss tangent, which is measured to be 0.15, 2.1 times lower at 20.7 MHz compared to PZT-5A. Compared to commonly used focusing acoustic transducers based on a curved surface or multi-element phased array, the planar SFAT is microfabricated with high precision, has small footprint, and can be easily operated without complex driving electronics.

The design of the SFAT has been verified through simulating the relative output acoustic pressure distribution in water with the finite-element method (FEM), with details described in Appendix A. Over the central vertical plane (Figure 1e), a strong focusing effect happens 5 mm above the transducer center with 790 *μ*m focal depth. On the focal plane (Z = 5 mm), the focal size is simulated to be 96 *μ*m (Figure 1f).

The transducer is microfabricated according to steps described in [34], in which the air cavities in the Fresnel acoustic lens are created through conformal deposition of a 4-*μ*m-thick Parylene-D film (Specialty Coating Systems Inc.) on a 3.5-*μ*m-thick sacrificial layer made of photoresist (AZ 5214-IR, Integrated Micro Materials). To create air cavities, the sacrificial photoresist is removed with acetone from patterned release holes (Figure 1d) on the Parylene layer. After wires are soldered on the top and bottom soldering pads, the release holes along with the soldered areas are sealed by another conformal deposition of thick (22 *μ*m) Parylene. The final Parylene thickness of 26 *μ*m (which equals to quarter wavelength) is chosen to ensure the highest acoustic energy transmission [35] from the transducer to the medium.

After fabrication, the transducer is packaged onto a laser-machined acrylic holder (Figure 2a). For an easy electrical connection, the wires soldered on the transducer are connected to a subminiature version A (SMA) adapter attached on the side of the holder, passing through a ferrite core tube (2673000701, Fair-Rite Products Corp.) that is used for shielding electromagnetic interference which may affect the operation of other equipment used for the tumor treatment.

### B. TUMOR TREATMENT SYSTEM SETUP

During transducer operation, a small portion of the input electrical power is dissipated in undesirable Joule and dielectric heating on and in the PZT, respectively, and raises the temperature of the transducer surface significantly, which increases the temperature on the treatment spot through heat conduction. We have confirmed that the temperature rise at the focal point is not from the heating effect due to acoustic energy. To reduce Joule heating from the series resistance of the electrodes, thick (10 *μ*m) silver electrode with low electrical resistivity is chosen along with a large soldering pad area (94.5 mm^2^) (inset of Figure 2a), which reduces the resistance to mere 5 mΩ, making Joule heating almost negligible. In addition, to dissipate the heat generated on the transducer from the PZT’s dielectric heating (due to its loss tangent), a compact water-cooling system is custom-built. As shown in Figure 2a, the backside of the transducer is attached to a small hollow water-cooling block made of nickel-coated copper (MCX Ram Block, Alphacool International GmbH) with thermally conductive paste (Kryonaut, Thermal Grizzly GmbH) having a high thermal conductivity of 12.5 W/(m · K). The transducer/water block assembly is held together by a clamping mechanism consisting of two laser-machined acrylic sheets with screws and nuts. On the acrylic sheet closer to the transducer surface, a 10 × 10 mm^2^ opening is cut out to let the ultrasound waves pass through and to store ultrasound transmission gel (Scan Ultrasound Gel, Parker Laboratories) which serves as the coupling medium between the SFAT and the treated tumor. The inlet and the outlet of the water-cooling block is connected to a refrigerated liquid circulator (Neslab NTE 740, Thermo Fisher Scientific Inc.) through two pieces of plastic tubing, forming a water-filled close loop. Driven by the pump of the circulator, the warmed water in the water-cooling block (due to the PZT’s dielectric heating) goes through the tubing and the refrigeration unit of the circulator, and then gets cooled down rapidly there. The cooled water is then pumped back into the water-cooling block, resulting in a closed loop in which the water temperature near the refrigeration unit is digitally controlled to be 8 °C, which is determined from thermal toxicity experiments described in the next section to avoid any potential thermal damage.

In order to treat a tumor volume (varying from 30 to 1,500 mm^3^) that is much larger than the volume of the focused ultrasound (about 4.2 × 10^−3^ mm^3^) generated by the transducer, a three-axis positioning and scanning system (Figure 2b) has been developed through modifying a commercial 3D printer (Alunar M508) whose print head is removed and replaced with a customized holding platform consisting of an acrylic sheet and several metal blocks (Figure 2c). The transducer holder can be fixed onto the holding platform with the SFAT facing down towards the heating platform, and with the inlet and outlet of the attached water-cooling block facing the back of the positioning system. As an aid for the alignment between the transducer and targeted tumor, also attached onto the holding platform are a low-power (<1 mW) laser diode (VLM-650–03 LPT, Quarton Inc.) fixed vertically facing down in a metal housing (Fixed Laser Mounting Stand, Adafruit Industries LLC) and its driving circuit (12 mm Coin Cell Breakout, Adafruit Industries LLC) powered by a coin cell battery (Figure 2c). Since the lateral position difference between the focused laser spot and the transducer center is fixed and can be calibrated, once the laser focal point is positioned on the target (tumor center) (Figure 2d), the transducer can be brought to the same position through moving it by the calibrated distances. In the positioning system, the movement in X and Z directions is realized by moving the holding platform, while Y direction movement is achieved through moving the heating platform where the mouse is placed and kept warm during treatment (Figure 2b and 2d).

### C. IN VIVO MICE TREATMENT PROTOCOL

Animal experimentation is conducted in accordance with the ethical federal guidelines mandated by the University of Southern California Institutional Animal Care and Use Committee (Protocol 20542, approved on April 29^th^, 2016). To test the effectiveness of SFAT treatment, the B16F10 melanoma tumor model [36] is grown in immune-competent C57/B6 living mice. Before injection, B16F10 cells are maintained in Dulbecco’s modified eagle medium (DMEM) supplemented with 10% v/v fetal bovine serum (FBS) and 1% penicillin-streptomycin in a humidified chamber at 37 °C under 5% CO_2_ and passed in fresh media every 2 to 3 days. Cells are harvested and diluted to 1 × 10^6^ cells/mL in media with 50% Matrigel by volume. On Day 0, each mouse is anesthetized and injected subcutaneously with 1 × 10^5^ cells in 100 *μ*L into a shaved area on one flank. The mice are then randomized either for treatment or as controls to remain untreated. Throughout the treatment period, all the mice are housed and maintained under identical conditions.

Treatment is administered immediately (<30 minutes) after injection on Day 0. For every seven days, treatment is repeated once per day on each animal for five consecutive days followed by two days without treatment. In each treatment, the treated animal with its tumor area freshly shaved is positioned on the heating platform (heated to 37 °C) of the positioning system while continuously receiving 1–4% isoflurane gas through a nose cone system to keep it sleeping (Figure 2d). Before the treatment, ultrasound transmission gel is applied into the shallow open window on the front acrylic sheet on the transducer surface to ensure good acoustic coupling between the transducer and the mouse skin. Then the SFAT with its top surface facing downward is attached to the movable holding platform. The surface tension between the ultrasound gel and the PZT/acrylic sheet keeps the gel from dripping down or flowing away. Next, with the aid of a laser diode, the transducer center is aligned to the center of the tumor (manually indicated by a skin marker) (Figure 2d). Then the laser is immediately turned off to avoid any potential heating effect, and the SFAT is lowered until the bottom of the front acrylic sheet (3 mm thick) reaches the top of the tumor, then raised 1.6 mm so that its focal point is at the top of the tumor, 0.4 mm below the skin surface (since the designed focal length is 5 mm and skin thickness is about 0.4 mm). During the treatment, the SFAT is mechanically scanned according to a pre-stored automatic scanning program written in G code, while simultaneously driven with pulsed sinusoidal electrical signals (with details mentioned in the next section) to produce focused ultrasound inside the tumor (Figure 2e). At each XY plane, the mechanical scanning pattern covers a circular area with 4.8 mm diameter, where the SFAT is raster-scanned at a speed of 2 mm/s between spots with 0.3 mm spacing, and stops at each spot for 0.4 second for treatment, for a total of 204 spots per plane (Figure 2f). The same scan pattern is repeated at six XY planes with 0.3 mm spacing in the Z direction, covering 1.5 mm height and taking 11.5 min in total. When the mechanical scanning program is finished, the electrical signal is turned off, and the SFAT is then moved up and to the side through pre-programmed movement. After that, the treated mouse is removed from the heating platform and allowed to recover from anesthesia in a cage.

During the treatment period, tumor growth is monitored at least twice per week until the volume reaches about 1,500 mm^3^ after eleven days of treatment, when animals are euthanatized and fresh tumors harvested. Two pieces of excised tumors from the control group are stored in PBS at 4 °C and are used within 12 hours in the *ex vivo* experiments for characterizing their acoustic properties (described in Appendix B). The remaining excised tumors are fixed in 4% paraformaldehyde overnight at 4 °C, embedded in paraffin, and 5-*μ*m-thick sections are prepared for histologic analyses. Sections are routinely stained with hematoxylin and eosin (H&E). In addition, immunohistochemistry (IHC) is performed with primary antibodies for Ki67 (MA5–14520, 1:50, Thermo Fisher Scientific Inc.) and cleaved caspase-3 (9964S, 1:200, Cell Signaling Technology Inc.), and then developed with DAB Poly Define Detection System (DS9800, Leica Biosystem GmbH) in BOND-III Automated IHC Stainer (Leica Biosystems). Whole slide images are scanned by VS120 Virtual Slide Microscope (Olympus Corp.) and analyzed in OlyVIA software (Olympus Corp.)

## III. RESULTS AND DISCUSSION

### A. CHARACTERIZATION OF TREATMENT PRESSURE

As it is difficult to directly measure the acoustic pressure within the tissue, the treatment acoustic pressure is estimated through FEM simulation along with measured parameters including the measured peak acoustic pressure in water, plus the acoustic attenuation coefficients and sound velocities within B16F10 melanoma tumor tissue and mouse skin.

**FIGURE 3.**
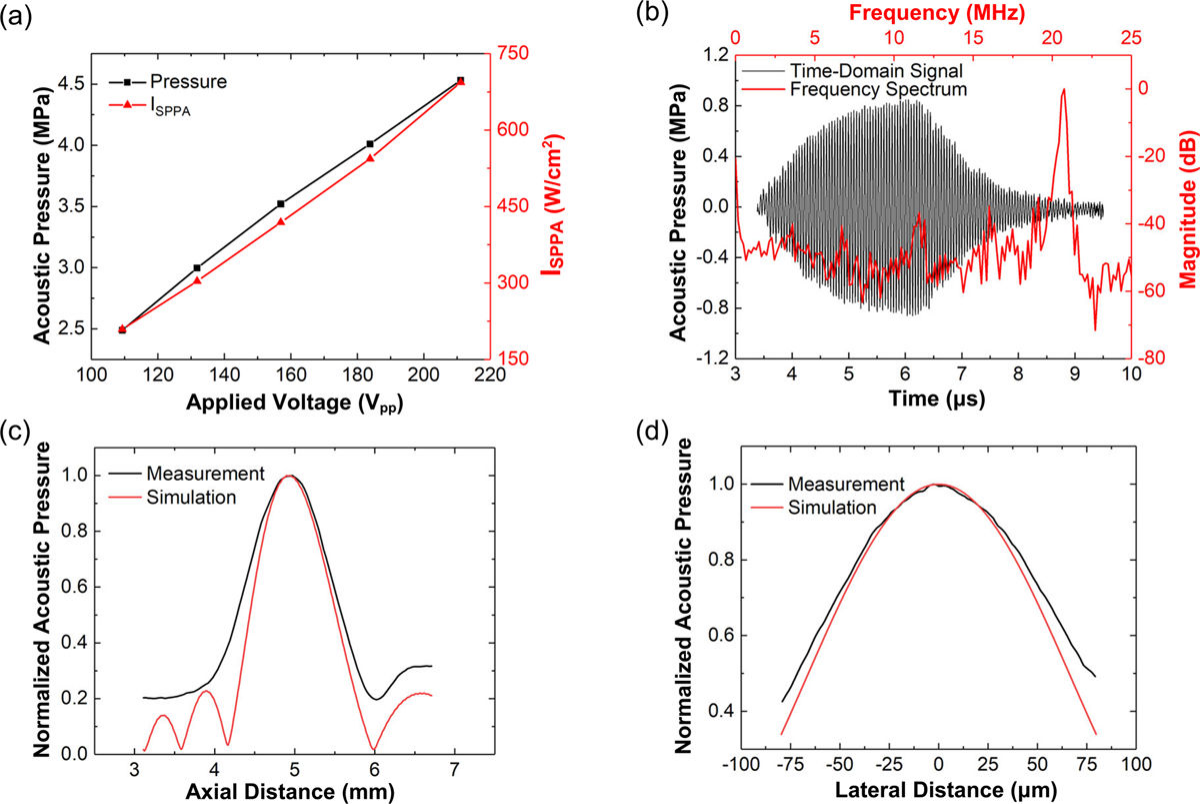
(a) Hydrophone measurement of the acoustic pressure (black) and the corresponding spatial-peak pulse-average intensity (I_SPPA_, red) at the focal point in water versus applied voltage. (b) Time-domain waveform of the acoustic pressure measured at the focal point (black, left and bottom axes) and the corresponding frequency spectrum (red, right and upper axes), with 60 cycles (2.9 *μ*s) of 20.7 MHz 40 Vpp sinusoidal voltage applied on the transducer. (c) (d) Measurement (black) and simulation (red) of normalized acoustic pressure in water (c) along the central vertical axis, and (d) along the central lateral axis on the focal plane.

A capsule-type hydrophone (HGL-0085, Onda Corp.) is used to characterize the acoustic intensity produced by the transducer. During measurement, the downward-facing hydrophone with its pre-amplifier is held by optical post clamps and optical posts (Newport Corp.) fixed on a five-axis high-precision movable stage consisting of two manual goniometric stages (GON-65L and GON-65U, Newport Corp.) and a three-axis motorized stage (OSMS26-XYZ, OptoSigma Corp.) The hydrophone is first scanned and aligned to the focal point of an unpackaged SFAT facing up in water (similar to the setup shown in Figure 9a but without tissue and ultrasound gel), through scanning the XYZ positions and adjusting the tilting orientations of the hydrophone in an iterative manner until the highest acoustic pressure can be measured. The hydrophone is then scanned along the central vertical axis (Figure 3c) and along the central lateral axis at the focal plane (Figure 3d) to measure the acoustic beam profiles of the FUS. During the hydrophone measurement (as well as the tumor treatment), a function generator (AFG-3252, Tektronix Inc.) is used to produce 20.7 MHz pulsed sinusoidal voltage signals, which are amplified by a power amplifier (75A250, Amplifier Research Corp.) and applied to the SFAT. For hydrophone tests, the driving pulse width is set to be 2.9 *μ*s (corresponding to 60 cycles of 20.7 MHz sinusoidal signals). An oscilloscope (MDO3014, Tektronix Inc.) is used to simultaneously monitor the applied voltage after a 40-dB voltage attenuator (100-SA-MFN-40, Bird Technologies) and the signal from hydrophone after a 20-dB pre-amplifier (AH-2010, Onda Corp.)

At the focal point where the highest acoustic pressure is observed, the measured acoustic pressure increases almost linearly as the applied voltage increases (Figure 3a) and reaches 4.53 MPa (when 211 V_pp_ is applied on the SFAT), which corresponds to I_SPPA_ of 693.7 W/cm^2^. The time-domain pressure signal measured at the focal point and its frequency spectrum are shown in Figure 3b. As can be seen from the latter, the highest spectrum peak is at the expected 20.7 MHz along with much smaller peaks generated from much weaker responses at the 1^st^, 3^rd^, 5^th^, and 7^th^ harmonic resonances of the PZT sheet. From the measured beam profiles, the focal length, focal depth, and focal diameter are 4.97 mm, 922 *μ*m (Figure 3c), and 104 *μ*m (Figure 3d), respectively, which are in good agreement with the simulated values. Similar tests are repeated with 3-mm-thick ultrasound transmission gel applied on the SFAT surface, and the measured acoustic pressure reduces only by 2% compared to the previous case without the gel, suggesting that the acoustic properties of water and ultrasound gel are very similar.

**FIGURE 4.**
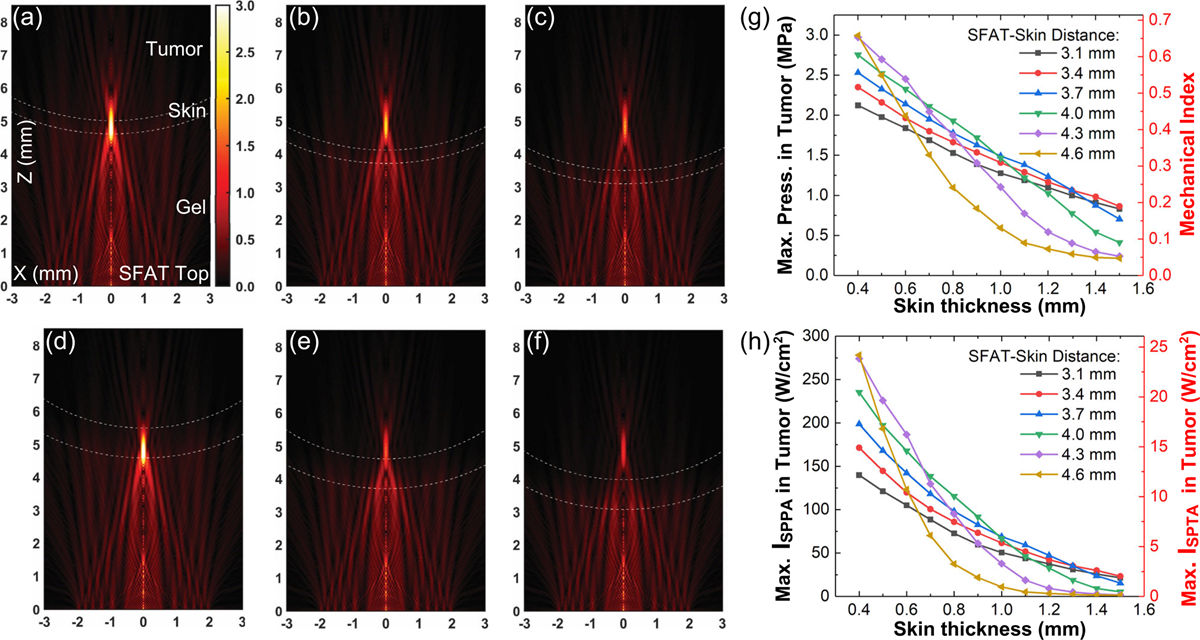
Simulated acoustic pressure distributions during treatment for 0.4 mm skin thickness, if the distance between the top of the SFAT and the bottom of skin is (a) 4.6 mm, (b) 3.7 mm, and (c) 3.1 mm; and similar simulations for 0.9 mm skin thickness, if the distance between the top of the SFAT and the bottom of skin is (d) 4.6 mm, (e) 3.7 mm, and (f) 3.1 mm; all sharing the same color bar in (a) with unit being MPa. (g) Simulated maximum acoustic pressure and corresponding mechanical index, and (h) simulated maximum I_SPPA_ and I_SPTA_ (with 1.45 ms pulse width and 60 Hz PRF) in tumor tissue during treatment versus different skin thicknesses as a function of SFAT-skin distances.

The attenuation coefficients and sound velocities within B16F10 tumor tissues and mouse skin has been measured through similar hydrophone tests (described in Appendix B), with results summarized in Table 2. Using the material properties from Table 2, we simulate the treatment acoustic pressure in tumor (described in Appendix A) which is modeled as an ellipsoid whose depth is 5 mm and diameter 11 mm, and the skin thickness is assumed to be uniform across the tumor, ranging from 0.4 to 1.5 mm in different simulations. The SFAT is aligned to the center of the tumor, and the distance between the SFAT and the bottom surface of the tumor skin is varied over 3.1, 3.4, 3.7, 4.0, 4.3, and 4.6 mm, which correspond to the cases where the SFAT is positioned in the center of the six XY scan planes (along the vertical Z direction) during treatment. From the simulation, we see that the maximum acoustic pressure within the tumor tissue varies with skin thickness and the SFAT-skin distance, and the focal zone extends up to about 1.5 mm into the tumor (Figure 4a to 4f). When skin thickness is 0.4 mm, the maximum treatment pressure varies from 2.1 to 3.0 MPa (Figure 4g), which corresponds to a mechanical index (MI, calculated by dividing the peak negative pressure in MPa by the square root of frequency in MHz [39]) of 0.46 to 0.66. The highest MI value of 0.66 is lower than the threshold value of 0.71 where cavitation may happen for short-pulse (a few cycles), low-duty-cycle (<1%) ultrasound [39], and much lower than the United States Food and Drug Administration (FDA) safety limit of 1.9 for diagnostic ultrasound [40].

### B. TEMPERATURE MEASUREMENT AND THERMAL TOXICITY TESTS

**FIGURE 5.**
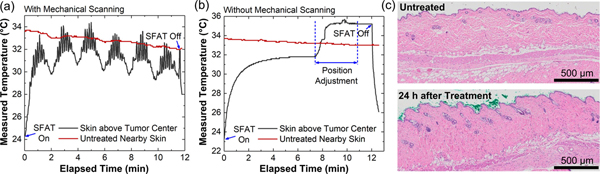
(a) Measured typical temperature change on the skin above the treated tumor center (grey) and on an untreated skin area nearby with no tumor underneath (red) during skin toxicity tests with the same experimental conditions of actual treatment except having the thermocouples. (b) Similar measurement as (a), but without mechanical scanning of the transducer, which is focused on the top skin surface above the tumor center. The position of the transducer is adjusted when the temperature saturates after 7.5 min of transducer actuation, for better alignment between the transducer and the thermocouple placed on the skin surface. (c) Representative histology images of untreated normal skin (upper panel) compared with skin harvested 24 hours after treatment (lower panel) in thermal toxicity experiments with a maximum temperature of 35.6 °C. No histologic changes are noted.

The device driving condition is determined through experiments in which skin temperature is monitored in real time to ensure there is no thermal damage. During the tests, we keep the same frequency, driving voltage, and pulse repetition frequency (PRF) of 20.7 MHz, 211 V_pp_, and 60 Hz, respectively, while varying the pulse width. With the same setup used for actual treatment, we attach one miniature k-type thermocouple (with a 0.8-mm-diameter tip) from a digital datalogging thermometer (HH506RA, Omega Engineering Inc.) onto the skin area above the tumor target to monitor the treatment temperature, while a separate thermocouple is positioned over untreated skin to measure the body temperature of the mouse. During treatment, the skin temperature slowly drops from about 34 to 32 °C as the mouse is being anesthetized and kept warm by the heating platform (Figure 5a). Before the treatment, the skin temperature in the center of the tumor is relatively low (24 to 25 °C) due to the cooled transducer surface and ultrasound gel. After the SFAT is turned on and being mechanically scanned, the temperature increases, then saturates and changes slightly during the treatment, as the distance between the SFAT and the thermocouple varies during mechanical scanning (the periodical temperature drops in Figure 5a happen when the ultrasound gel cooled by the relatively cold non-active surface area of SFAT reaches the thermocouple). From the experiments, a pulse width of 1.45 ms is chosen so that the maximum skin temperature in the treatment area remains below 35 °C throughout the 11.5 min treatment, which is less than 1 °C higher than the measured normal body temperature (Figure 5a).

In another test, to further evaluate any possible thermal effect, a similar measurement is carried out without scanning the transducer. Due to the difficulty to directly monitor the temperature rise within the tissue, the SFAT is aligned to the skin surface above the tumor center to evaluate the ultrasound-induced temperature rise, and is actuated with the same driving conditions as those used during treatment. When the measured temperature on the skin surface in the treatment region stabilizes after 7.5 min of device operation, the position of the SFAT is adjusted in the X, Y, or Z direction by 0.1 mm each time, while the skin temperature is being monitored for at least 5 s without movement to observe the effect of position adjustment. This scanning process of 0.1 mm movement followed by temperature monitoring without movement is repeated until the skin temperature reaches its maximum and restabilizes. Since the initial laser-guided alignment between SFAT and the target area is already very good, the position adjustment process takes less than 3 min, with the maximum skin temperature being 35.6 °C (Figure 5b).

With this temperature, the estimated cumulative equivalent minutes at 43 °C (CEM_43_, the accepted metric for thermal dose assessment, which estimates the equivalent exposure time at 43 °C for a thermal exposure of a certain temperature for a given time) for an 11.5 min treatment is only 24 ms, which is much less than a thermal dose which would be expected to cause any thermal damage [41].

Thermal toxicity tests on normal mouse skin with the same treatment condition and duration are also conducted to examine the potential thermal damage on normal tissues. The 11.5 min treatment with a maximum temperature of 35 °C measured directly in the treated area does not seem to cause any lasting visible or microscopic lesions. No histologic effect is observed on normal, shaved mouse skin in non-tumor-bearing animals 24 hours after the treatment (Figure 5c).

With a pulse width of 1.45 ms, for an estimated treatment pressure of 2.1 to 3.0 MPa (assuming a skin thickness of 0.4 mm), the calculated I_SPPA_ and I_SPTA_ are 136.8–279.1 and 11.9–24.3 W/cm^2^ (Figure 4h), respectively, which are about an order of magnitude lower than those used in thermal-based HIFU treatment. Compared to the FDA safety limits for diagnostic ultrasound (I_SPPA_ < 190 W/cm^2^, I_SPTA_ < 720 mW/cm^2^, MI < 1.9) [40], our values are close in I_SPPA_, much higher in I_SPTA_, and much lower in MI. Thus, we define our treatment intensity to be “medium intensity” when compared to HIFU and diagnostic ultrasound.

### C. TREATMENT RESULTS

**FIGURE 6.**
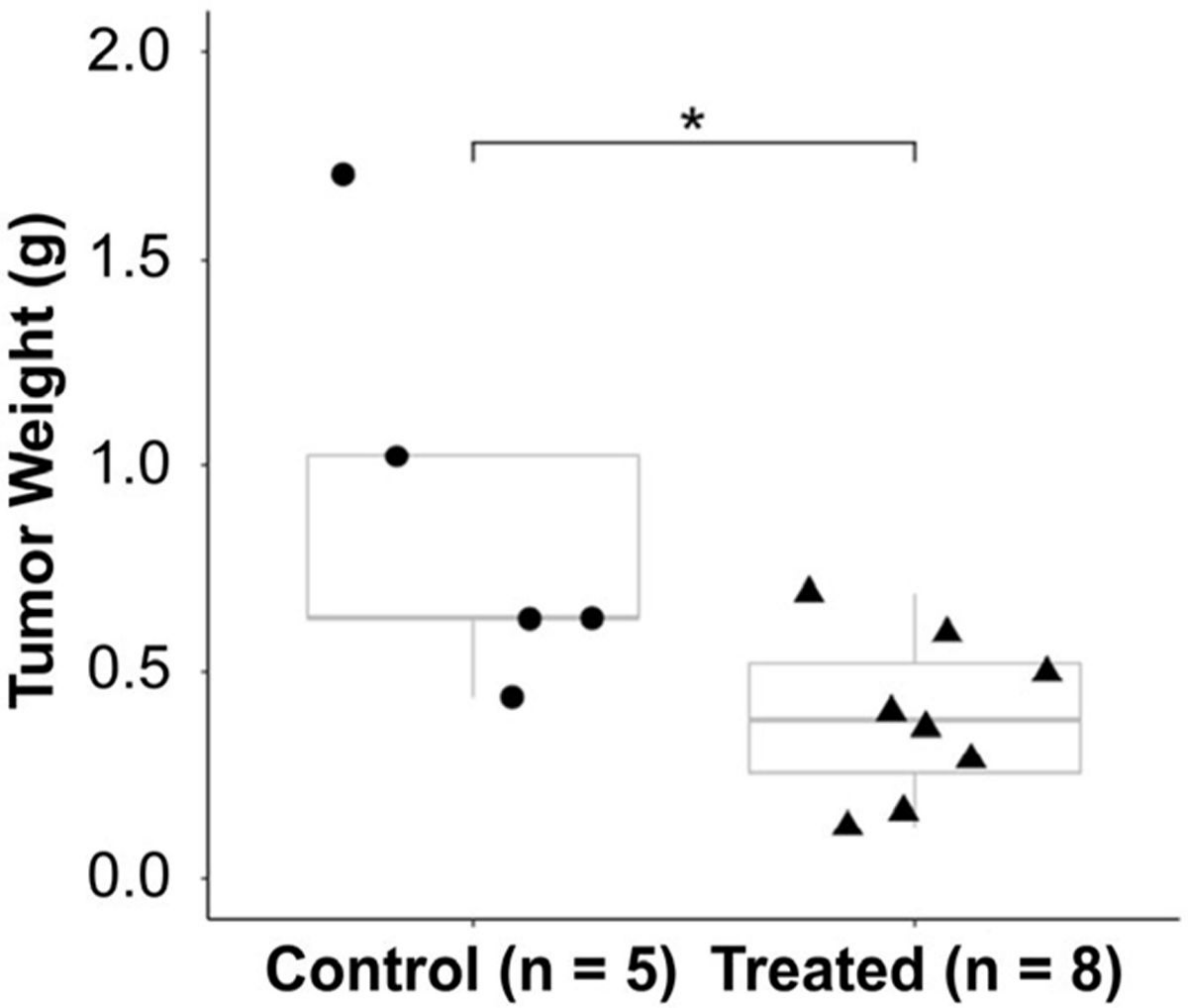
Tumor weights of control (n = 5) and treated (n = 8) tumors shown in boxplots (*: student’s t-test p < 0.05).

The tumor weights measured at the end of the treatment are summarized in Figure 6. We observe a significant difference in mean tumor weights according to treatment assignment. Specifically, the mean ± standard deviation weights of the treated versus the untreated tumors are 390 ± 200 mg (n = 8) versus 885 ± 506 mg (n = 5), respectively (student’s t-test, p = 0.03).

**FIGURE 7.**
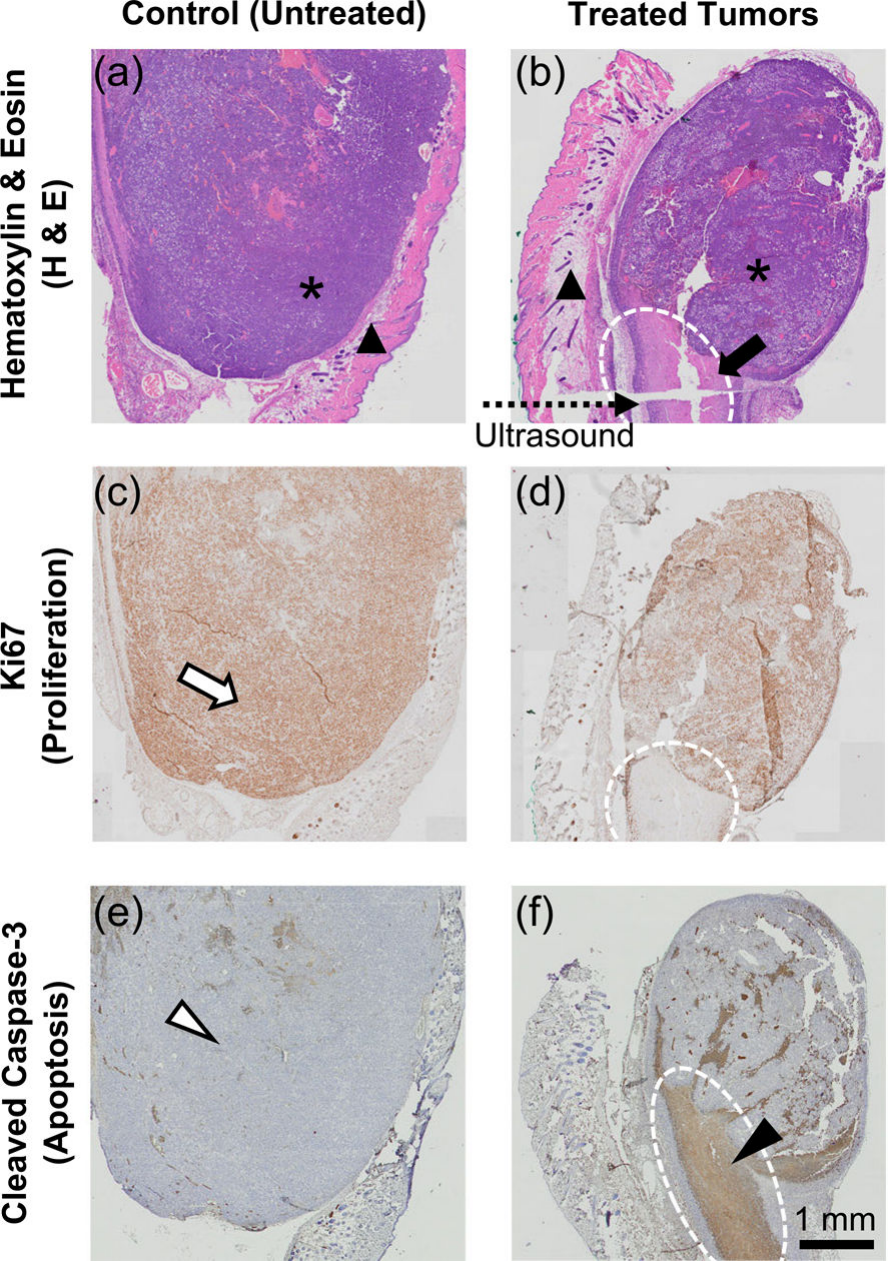
Representative cross-sectional histologic images of control and treated B16F10 tumors, showing matched sections representing control (untreated) and ultrasound-treated tumors that are stained for H&E ((a) and (b)), Ki67 (an indicator of cell proliferation, (c) and (d)), and cleaved caspase-3 (an indicator of cell apoptosis, (e) and (f)). *: viable tumor; ▲: normal skin overlying tumor; solid arrow and solid arrowhead: area of necrosis and apoptosis; open arrow and open arrowhead: large area of proliferating cancer cells without apoptosis; dotted arrow: direction of applied ultrasound; dotted white ellipses: ultrasound treatment areas.

**FIGURE 9.**
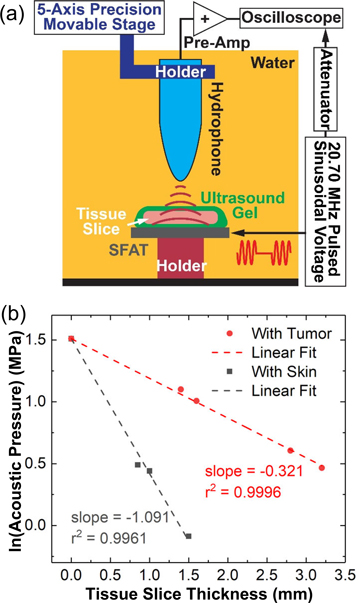
(a) Cross-sectional diagram of the hydrophone measurement setup with a piece of tissue slice attached to the top of the SFAT with ultrasound transmission gel. (b) Natural logarithm of the measured acoustic pressure at the focal point when tumor (grey dots) or skin (red dots) slices of different thicknesses are placed on the SFAT, with linear fitting lines whose slopes are equal to the attenuation coefficients in the tissues (unit: np/mm).

Histologic analysis is used to identify the effects of focused ultrasound treatment on tumors. While areas of viable cancer cells are readily found in both treatment and control groups (indicated by * in Figure 7a and 7b), the tumor area is smaller in the treated tumors. Moreover, in the expected treatment areas of the treated tumors, large areas of necrosis are identified, which can be seen in a large pale area of necrotic cells lacking nuclear detail (solid arrow in Figure 7b), the volume of which is similar to the expected treatment volume. Cells in the same area also exhibit strong expression of cleaved caspase-3, suggesting ongoing cell apoptosis (solid arrowhead, Figure 7f). In contrast, the untreated tumor exhibits a large continuous area of highly proliferative cancer cells indicated by strong Ki67 expression (open arrow, Figure 7c) without cleaved caspase-3 expression (open arrowhead, Figure 7e). In all cases, no damage is found in the skin area positioned directly under the transducer during the treatment (solid triangles in Figure 7a and 7b).

### D. DISCUSSION

In an *in vivo* treatment using the B16F10 cell model on mice, selective cancer treatment has been achieved with pHFMIFU generated by an SFAT, when the heat generated during treatment is too small to cause any damage. Compared to other works on ultrasound cancer treatment (summarized in Table 3), our technology effectively and selectively kills cancer cells without relying on external agents or high temperature, and the risk of inertial cavitation is low. Moreover, the generated high-frequency focused ultrasound has very fine spatial resolution, which makes it better suited for applications where the tumor size is small or when the treated area is adjacent to critical tissues.

The selective killing effect of pHFMIFU can potentially be contributed to several factors. Previously, we suggested that this selectivity might be a result of the disorganized cytoskeletal structure observed in cancer cells [27], [42], resulting in a reduced cell stiffness [42], [43], making them much easier to deform under mechanical stress, and thus easier to be damaged. It has been demonstrated in *in vitro* experiments that the cytoskeleton could be disrupted by very low intensity (290 kPa) of ultrasound at 1 MHz [44]. Due to the less organized cytoskeletal structure of cancer cells, the acoustic intensity threshold for permanent damage on cancer cells is likely lower compared to that for benign cells, which has been confirmed by our previous *in vitro* experiments [27]. Another possible cause for this selectivity is the different natural mechanical resonant frequencies of cancerous cells and healthy cells, resulted from their differences in material properties (such as stiffness) and cell geometries (such as nuclei size) [45], [46]. As a result, cancer and normal cells will effectively react to ultrasound of different frequencies.

The exact mechanism of damage from our non-thermal pHFMIFU treatment is so far unclear, but there are several theories available to explain the effect. According to the low MI (<0.66), the chance of inertial cavitation is small. However, with the relatively long pulse width of 1.45 ms, stable cavitation may be induced, in which microbubbles are formed and periodically oscillates without collapsing. These oscillating microbubbles can move at high speed in response to ultrasound-induced acoustic radiation force [47], generate microstreaming motion around them [48], and scatter acoustic waves [49], causing bioeffects such as increased cell membrane permeability and perturbed cytoskeleton structure [50], which can cause destructive damage to cells. Apart from cavitation, ultrasound itself can induce damage through acoustic radiation force which may produce displacements of cells to generate shear strain [51], or through generating acoustic microstreaming flow inside or around cells [52]. In another theory, the acoustic waves could react on the lipid bilayer cell membrane, causing it to expand and contract, thus transforming the oscillating acoustic pressure waves into smaller-scale intracellular deformations [53]. In our future work, we plan to carry out experiments to elucidate the underlying mechanisms of this selective cancer treatment, using advanced imaging and analyzing tools. For example, the ultrasound-induced cell death during and after treatment could be monitored using ultrasound imaging [54], MRI [55], single photon emission computed tomography (SPECT) [56], or positron emission tomography (PET) [56]. On the cell level, scanning electron microscopy (SEM) could be used to examine the ultrasound-induced damage [53]. In addition, the real-time temperature rise within the tumor during treatment could be measured contactless with ultrasound imaging [57], MRI [5], [6], or thermal infrared imaging. Moreover, the ultrasound-induced cavitation could be monitored through measuring the acoustic emission spectrum with another wide-band transducer [58], [59]. On top of these, the influences of different treatment conditions (such as operating frequency, acoustic pressure, acoustic intensity, pulse width, PRF, and treatment duration) on the effectiveness of treatment will be evaluated through design of experiments (DOE) using techniques such as the Taguchi method [60] to find the optimized conditions for realizing the best selective therapeutic effects on tumors with shorter treatment time and lower acoustic power.

While we observed a decrease in the average tumor weight in the treated group, the results are not uniform or consistent across all tumors, and we do not see a complete destruction of cancer cells in the treated animals, possibly due to the following reasons. The first reason is the discrepancy between the treated volume and the actual tumor volume. To avoid an impractically long treatment time, we limit the number of treatment spots, covering only a small portion of the total treatment volume. With the current scanning pattern, the total effectively treated volume consisting of all the treatment spots (where the transducer stops for 400 ms) covers only 12.2% of the total treatment volume. Even with the inclusion of the scanned volume (where the transducer is moved at a relatively fast speed of 2 mm/s between treatment spots), the value increases only to 51.0%, suggesting a large volume of tumor being left untreated (Figure 2f). Moreover, as the treatment target is established immediately prior to each activation, the shape and extent of the total treatment volume is fixed once treatment is started, and remain the same over the entire course of the experiment. During the later phase of the tumor growth experiment, tumor diameters of 5–12 mm are observed in some of the treated tumors, but the treatment pattern constrained by the 4.8 mm circular raster pattern remains constant. Also, the treatment volume can be affected by the respiratory and involuntary motion (up to several millimeters) from anesthetized live mice, especially considering the short treatment time on each treatment spot (corresponding to only 24 pulses per spot). Finally, the shaved mouse skin is not perfectly uniform over the tumor-bearing areas which may result in inhomogeneous acoustic doses delivered to each tumor. To ensure better treatment consistency in the future, we will realize a full treatment coverage of tumors without increasing the treatment time through modifying the Fresnel lens design to increase the focal depth and size of the ultrasound [61]. The effective treatment volume can also be increased by having an array of transducers, which can also enable electrically controlled ultrasound beam scanning without mechanical movement. In addition, a customized scanning pattern to better fit the tumor shape can be implemented through 3D shape scan of tumors.

## IV. CONCLUSION

To further confirm the non-thermal, selective killing effects of high-frequency focused ultrasound that was previously demonstrated in *in vitro* experiments involving monolayers and spheroids of cancer cells, this study examines the therapeutic effects of pulsed (60 Hz PRF, 1.45 ms pulse width) high-frequency (20.7 MHz) focused ultrasound with medium-intensity (peak pressure < 3.0 MPa, I_SPPA_ < 279.1 W/cm^2^, I_SPTA_ < 24.3 W/cm^2^, MI < 0.66) in an *in vivo* subcutaneous B16F10 melanoma tumor growth model in mice. The ultrasound is generated by an SFAT with Fresnel air-cavity lens designed for 5 mm focal length, with a measured focal diameter and focal depth of 104 and 922 *μ*m, respectively. A compact three-axis positioning and scanning system has been developed to realize automatic mechanical scanning of the transducer to cover a larger cylindrical treatment volume with 1,224 treatment points in 11.5 min with 400 ms duration per spot. A close loop water-cooling system is custom-built for dissipating non-acoustic heat from the transducer surface. Throughout the treatment, the skin temperature in the treated area is kept below 35.6 °C, which is too low to generate any thermal damage. After eleven days of ultrasound treatment, the treated tumors have significantly less weight compared to the untreated ones, and histologic analyses have revealed cell necrosis and apoptosis in the expected treated area underneath the skin. In addition, no damage has been detected in normal skin or tissues in the treatment or thermal toxicity tests, suggesting a successful non-thermal, selective tumor treatment.

In contrast to low-frequency high-intensity ultrasound, our *in vivo* experiment demonstrates the potential use of pHFMIFU as a new tool for selective cancer treatment with much better spatial resolution, in regions where it is critical to keep the surrounding normal cells and tissues undamaged from the cancer treatment. The potential mechanisms of this selective killing effect as well as the possible causes of and solutions to the variation in treatment results are analyzed and proposed.

## Figures and Tables

**FIGURE 8.**
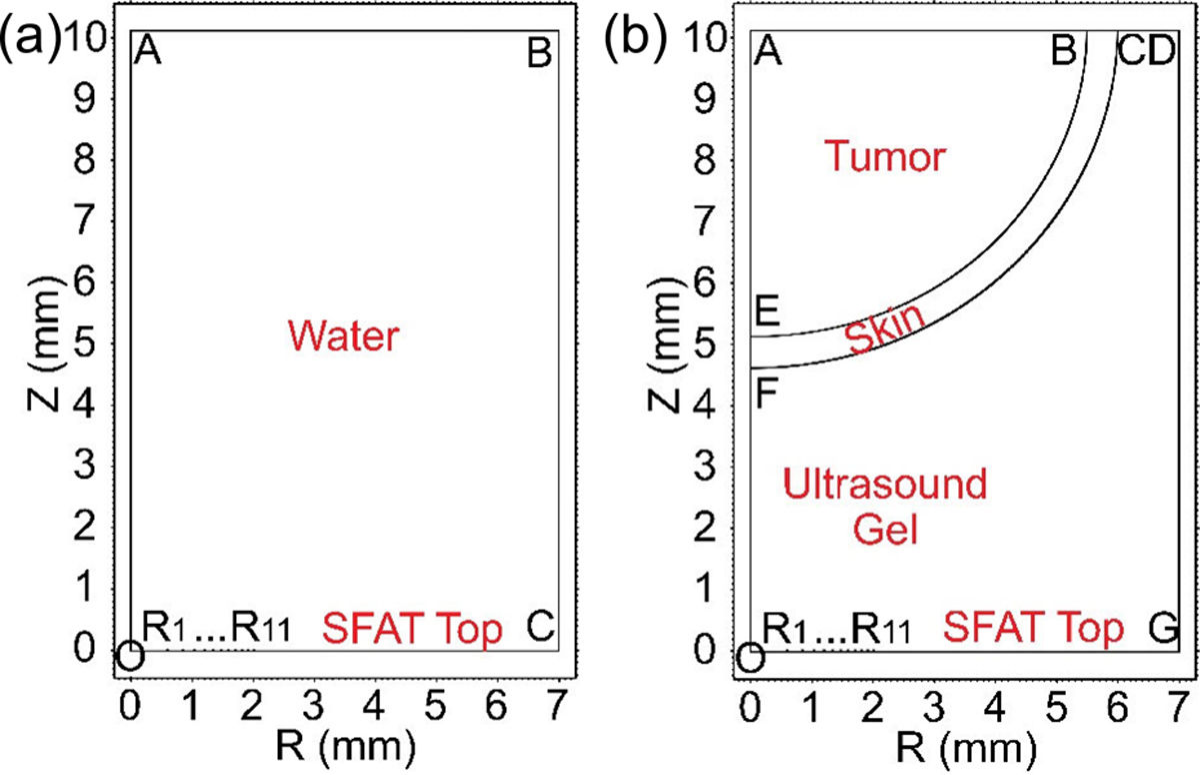
Defined 2D simulation areas with axisymmetry for simulating output acoustic pressure distribution (a) when the SFAT is immersed in water and (b) when ultrasound transmission gel is applied between the SFAT and a tumor with 0.5-mm-thick skin (with the distance between SFAT’s top surface and the skin’s bottom surface being 4.6 mm).

**TABLE 1.** Design parameters of the transducer used for *in vivo* tumor treatment.

**Substrate Material**	**Transducer Dimensions**	**Air Cavity Height**	**Parylene Thickness**
PZT-4 (DL-47)	16 × 16 × 1 mm^3^	3.5 μm	26.0 μm

**Electrode Material**	**Working Frequency**	**Simulated Focal Length**	**Simulated Focal Diameter/Depth**
10-μm-thick silver	20.7 MHz (9^th^ harmonic)	5 mm	96 μm/790 μm

**Boundary Radii of Non-Air-Cavity Fresnel Circle and Rings (Inner, Outer in μm)**		

1^st^ circle	2^nd^ ring	3^rd^ ring
0, 595	843, 1035	1197, 1340
4^th^ ring	5^th^ ring	6^th^ ring
1471, 1591	1704, 1811	1912, 2009

**TABLE 2.** Material properties used in the simulation of the treatment acoustic pressure.

Material	Thickness (mm)	Mass Density (kg/m^3^)	Sound Velocity (m/s)	Attenuation Coefficient (np/mm)

B16F10 Tumor	5 (11 in diameter)	1060*	1521**	0.321**
Mouse Skin	0.4–1.4	1060*	1558**	1.091**
Ultrasound Gel***	NA	1000	1480	0.110

* The average density of soft tissues [37]

** From measurement (described in Appendix B)

*** Used the values for water [38] due to similar acoustic properties

**TABLE 3.** Comparison between this work and related works about ultrasound-based cancer treatment*.

References	Operating Frequency	Intensity (I)/Pressure (P)/Energy(E)	Pulse Conditions	Focal Diameter/Depth of Focus	Experiment Objects	External Agents Used	High Temperature/Inertial Cavitation	Selectivity

[2]	0.8 and 1.6 MHz	I_SPPA_ = 5,000–20,000 W/cm^2^	NA	1.3 mm/9.8 mm	Human patients with solid carcinomas (*in vivo*)	None	Yes/NA	No
[3]	4.0 MHz	I_SPPA_ = 1,260–2,000 W/cm^2^	4 s PW 25% DC	NA	Human patients with prostate cancer (*in vivo*)	None	Yes/Y es	No
[9]	0.7 MHz	P = 14.0 MPa	300 Hz PRF 5.71 μs PW	5 mm/10 mm	Human liver tumors in pigs (*in vivo*)	None	No/Yes	No
[10]	1.0 MHz	P > 30.0 MPa	100 Hz PRF 1–2 μs PW	NA	N1-S2 HCC liver tumor in rats (*in vivo*)	None	No/Yes	No
[21]	4.0 MHz	E = 24 J/spot	Continuous for 3 s/spot	3 mm/12 mm	Co-cultures of cancerous and normal cells (*in vitro*)	Gold/magnetic nanoparticles	No/NA	Yes
[22]	0.94 MHz	P = 3.0 MPa	10 Hz PRF 0.19% DC 3 s on and 9 s off	NA	Subcutaneous sarcoma S-180 cells in mice (*in vivo*)	Intravenously injected microbubbles	No/Possible	Yes
[23]	2.0 MHz	I_SPPA_ = 0.33 W/cm^2^	Continuous for 2–4 min	NA	Monolayers of cancerous and normal cells (*in vitro*)	None	No/Possible	Yes
[25]	0.50–0.67 MHz	P< 1.2 MPa I_SPTA_ < 5W/cm^2^	PW > 20 ms 10% DC	NA	Suspensions of cancerous and normal cells (*in vitro*)	None	No/Yes	Yes
[26]	20.0 MHz	E = 0.6–1.2 J/shot	150 ms/shot, 1–2 s between shots	NA/1.1–1.7 mm	Human patients with actinic keratosis and skin cancers (*in vivo*)	None	Yes/NA	No
[27]	17.3 MHz	I_SPPA_ < 8.88 W/cm^2^	1 s PW 50% DC	0.1 mm/0.8 mm	Monolayers of cancerous and normal cells (*in vitro*)	None	No/No	Yes
[28]	17.3 MHz	I_SPPA_ < 15.14 W/cm^2^	60 Hz PRF 58 μs PW	0.16 mm/0.7 mm	Spheroids of cancerous and normal cells (*in vitro*)	None	No/NA	Yes
This work	20.7 MHz	P < 3.0 MPa I_SPPA_ < 279.1 W/cm^2^ I_SPTA_ < 24.3 W/cm^2^	60 Hz PRF 1.45 ms PW 0.4 s/spot	104 μm/922 μm	Subcutaneous B16F10 melanoma tumors in mice (*in vivo*)	None	No/Low risk (MI < 0.66)	Yes

* Abbreviations: PW: pulse width; DC: duty cycle; PRF: pulse repetition frequency; I_SPPA_: spatial-peak pulse-average intensity; I_SPT_ : spatial-peak temporal-average intensity, MI: mechanical index.

## APPENDIX A

### SETTINGS FOR ACOUSTIC PRESSURE SIMULATION

The acoustic pressure distribution of the SFAT is simulated with the finite-element method (FEM) using the Pressure Acoustics module of COMSOL Multiphysics (COMSOL Inc.) at 20.7 MHz. For simplicity, only the volume above the transducer is considered, and the acoustic waves coming from the transducer are modeled with normal displacement boundary conditions defined on the non-air-cavity Fresnel circle and rings. To save computation time and memory, two-dimensional (2D) axial symmetry is defined, where only a half of the volume cross-section is modeled (Figure 8). All 2D simulation plots presented in this paper are created through mirroring the simulated data along the central vertical axis (R = 0). For the FEM analysis, a free triangular mesh is used with a maximum element size of 70.6 *μ*m (equal to 1/10 of the wavelength in water). For simplicity, all materials modeled in the simulations are assumed to have isotropic and homogeneous material properties.

For simulation results shown in Figure 1e and 1f, the boundary conditions are defined as follows (with material and boundary notations defined Figure 8a): (1) axial symmetry for AO; (2) perfectly matched boundaries (no reflection) for ABC; (2) sound hard boundaries (air interfaces) for R_1_R_2_, R_3_R_4_, R_5_R_6_, R_7_R_8_, R_9_R_10_; (3) acoustic impedance of PZT for R_11_C; (4) normal displacement (non-air-cavity circle and rings) for OR_1_, R_2_R_3_, R_4_R_5_, R_6_R_7_, R_8_R_9_, R_10_R_11_.

For simulation results shown in Figure 4a to 4f, the boundary conditions are defined as follows (with material and boundary notations defined Figure 8b): (1) axial symmetry for AEFO; (2) perfectly matched boundaries (no reflection) for ABCD; (3) sound hard boundaries (air interfaces) for R_1_R_2_, R_3_R_4_, R_5_R_6_, R_7_R_8_, R_9_R_10_, DG; (4) acoustic impedance of PZT for R_11_G; (5) normal displacement (non-air-cavity circle and rings) for OR_1_, R_2_R_3_, R_4_R_5_, R_6_R_7_, R_8_R_9_, R_10_R_11_, with displacement amplitude normalized from hydrophone measurements using the peak pressure at the focal point.

## APPENDIX B

### CHARACTERIZATION OF ACOUSTIC ATTENUATION COEFFICIENTS AND SOUND VELOCITIES WITHIN MICE SKIN AND TUMOR TISSUE

Freshly excised tissues are dissected with a scalpel to produce tumor and normal skin samples of varying thicknesses. With the hydrophone measurement setup shown in Figure 9a, a tumor or skin slice is attached onto the SFAT’s top surface with a thin (<1-mm-thick) layer of ultrasound transmission gel. In each measurement involving a tumor/skin slice, the acoustic pressure at the focal point is measured with the hydrophone. Taking the peak acoustic pressure measured in water with no tissue/skin above the transducer to be *P*_0_ (measured to be 4.53 MPa as shown in Figure 3a), the attenuated focal-point pressure *P*_*atten*_ after passing through a thin layer of tissue with thickness *d* and attenuation *α* can be expressed with

(1)
$$P_{atten\;}=P_{0}\times e^{-\alpha d},$$

For simplicity, in (1), we ignore the acoustic loss due to reflection from the tissue slices (which is small, since tissues have similar acoustic impedances to that of water/ultrasound gel). Also, we approximate the path-length from anywhere in the small transducer active area to the focal point to be equal to the focal length (paraxial approximation). By taking the natural logarithm on both sides of (1), we have

(2)
$$ln\left(P_{atten}\right)=\ln\left(P_{0}\right)-\alpha d,$$

where ln(*P*_0_) is a constant. Thus, by plotting ln(*P*_*atten*_) versus *d*, we can extract the attenuation coefficient (*α*) through a linear fitting. From Figure 9b, the attenuation coefficients in tumor and skin in the mouse are estimated to be 0.321 and 1.091 np/mm at 20.7 MHz, respectively.

Additionally, to estimate the sound velocities in tumor or skin tissues (*c*_1_), we measure the focal length (*F*) and the time-of-flight for the first ultrasound beam from the transducer center to arrive at the hydrophone (*t*_1_) after passing through a tissue with thickness *d*. Assuming that the sound velocities in the ultrasonic gel and water are the same (*c*_0_ = 1480 m/s [38]), we have

(3)
$$t_{1}=d/c_{1}+(F-d)/c_{0},$$

from which we get

(4)
$$c_{1}=c_{0}d/\left(c_{0}t_{1}-F+d\right).$$

According to the measurements, the average (n = 3) measured sound velocities in the tumor and skin are 1,521 and 1,558 m/s, respectively.
